# Safe Path Planning Algorithms for Mobile Robots Based on Probabilistic Foam

**DOI:** 10.3390/s21124156

**Published:** 2021-06-17

**Authors:** Luís B. P. Nascimento, Dennis Barrios-Aranibar, Vitor G. Santos, Diego S. Pereira, William C. Ribeiro, Pablo J. Alsina

**Affiliations:** 1 Department of Computer Engineering and Automation, Federal University of Rio Grande do Norte, Natal 59078-970, Brazil; diego.pereira@ifrn.edu.br (D.S.P.); willcr@ieee.org (W.C.R.); 2 Federal Institute of Rio Grande do Norte, Parnamirim 59143-455, Brazil; vitor.gaboardi@ifrn.edu.br; 3 Electrical and Electronics Engineering Department, Universidad Católica San Pablo, Arequipa 04001, Peru; dbarrios@ucsp.edu.pe

**Keywords:** mobile robot, path planning, bubbles, probabilistic foam, safety, A* algorithm

## Abstract

The planning of safe paths is an important issue for autonomous robot systems. The Probabilistic Foam method (PFM) is a planner that guarantees safe paths bounded by a sequence of structures called bubbles that provides safe regions. This method performs the planning by covering the free configuration space with bubbles, an approach analogous to a breadth-first search. To improve the propagation process and keep the safety, we present three algorithms based on Probabilistic Foam: Goal-biased Probabilistic Foam (GBPF), Radius-biased Probabilistic Foam (RBPF), and Heuristic-guided Probabilistic Foam (HPF); the last two are proposed in this work. The variant GBPF is fast, HPF finds short paths, and RBPF finds high-clearance paths. Some simulations were performed using four different maps to analyze the behavior and performance of the methods. Besides, the safety was analyzed considering the new propagation strategies.

## 1. Introduction

Path planning is one of the most important problems in autonomous robot navigation, and it has been discussed in the scientific community since the 1980s [1,2,3]. The issue of planning is particularly relevant because it is almost a requirement for an autonomous mobile robot to perform a motion from an initial to a goal position while avoiding possible collisions in an environment with obstacles and narrow passages [4].

Initially, researches were focused on the development of path planners based on Roadmaps [5], Cell Decomposition [6], and Potential Field [7]. Due to the so-called curse of dimensionality due to high-dimensional configuration spaces, the researchers were motivated to develop sampling-based approaches for path planning, which usually use few computer resources and have been widely used for this sort of problem [8,9,10,11,12]. The most important sampling-based (probabilistic) path planning methods are Rapidly-Exploring Random Tree (RRT) [13,14] and Probabilistic Roadmaps (PRM) [15]. Further, Karaman and Frazzoli [12] have proposed the methods RRT* and PRM*, two of the most successful variants of RRT and PRM, respectively.

Most of the path planning methods are mainly dedicated to generate optimal paths or find feasible paths with reduced execution time [16]. Nevertheless, it is also essential to ensure safety for a robot when moving in unstructured environments [17]. Thus, planning paths sufficiently far from the obstacles is vital for most applications [18].

There are path planners that primarily intend to generate paths with high clearance from obstacles. The path planning methods based on the Voronoi diagram [3,19,20] are approaches that guarantee clearance along the paths. However, they are not practical for problems involving many degrees of freedom (DOFs) and many obstacles [21], due to the explicit computation of the configuration space obstacles.

Paliwal and Kala [22] presented a goal-oriented path planning algorithm that provides high clearance from obstacles by propagating circles in the free configuration space. Nevertheless, this method is limited to maps where the clearance is at least twice the robot, i.e., environments with large passageways. In [23], an improved A* algorithm is proposed to plan paths as far away as possible from the obstacles. In this approach, the environment needs to be subjected to improved modeling using a danger coefficient based on the distance between the robot and the obstacles. However, this approach may be challenging for high-dimensional spaces.

On the other hand, several strategies that intend to increase the clearance from obstacles along a given path are already planned, such as [21,24,25,26]. In [27], the free configuration space exploration is guided by a heuristic function based on a safety measure called Danger Field. In [28], Quinlan and Khatib proposed a framework to deal with collision-free motion using structures called Elastic Strips that can deform a path known *a priori*. However, safety was not addressed explicitly.

**Figure 1:**
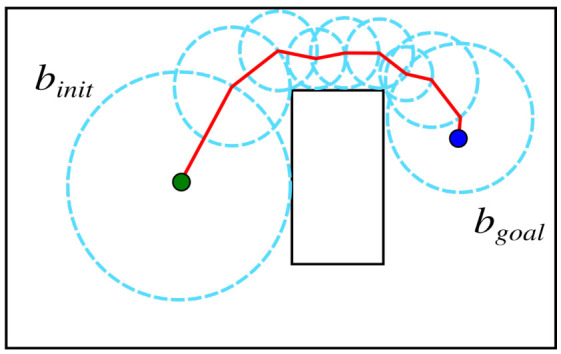
Solved path planning. Extracted rosary and found path (red line).

A sampling-based path planner called the Probabilistic Foam Method (PFM) was proposed in [29] with the main feature of ensuring an obstacle-free region for safe maneuverability. The safe region is provided by a structure called bubble, which has an *n*-ball shape, and represents a subset of the free space. A set of bubbles propagate through the free space from the initial configuration towards the goal configuration in a tree structure called probabilistic foam. The resulting path for this approach is extracted from a sequence of overlapped bubbles, called the rosary, which bounds the path and guarantees safe motions, also providing safety constraints for adjustments, such as path smoothing [30]. The rosary is illustrated in Figure 1.

In [31,32], we used the PFM to compute a set of safe configurations for a lower limb active orthosis to transpose a simple obstacle. The main contribution of [31] was the modeling of new bubbles inspired by the concept of *Bubble of free space*, proposed in [33], which enabled PFM to solve robotic problems with many degrees of freedom. In [34], we presented a variant of PFM called Goal-Biased Probabilistic Foam (GBPF). In this variant, we proposed a new foam propagation approach inspired by goal-biased RRT tree growing, which returns paths with the convenient trade-off between finding short paths through narrow passages in the map and keeping the clearance from obstacles. Additionally, GBPF is fast and usually computes a few bubbles.

Beyond the already known algorithms PFM and GBPF, in this paper, we present two new variants of Probabilistic Foam: Radius-biased Probabilistic Foam (RBPF) and Heuristic-Guided Probabilistic Foam (HPF). The variant RBPF is capable of finding paths with high-clearance areas in the map, and HPF converges fast towards to the goal configuration, generating short paths. For the probabilistic foam-based planners, where the rosary bounds the path, it is necessary some metrics to measure the clearance from the obstacles considering the influence of the bubbles. Thus, we also propose in this work some metrics to evaluate the four algorithms.

The remainder of the paper is organized as follows: Section 2 describes the original PFM, and a new approach to determine one of its parameters is proposed. In Section 3, we present the GBPF algorithm [34] and propose two new variants of PFM—RBPF and HPF. Section 5 presents simulation results and discussions. Finally, we present some conclusions and future works in Section 6.

## 2. The Probabilistic Foam Method

Probabilistic Foam Method (PFM) is a sampling-based path planning algorithm, initially proposed in [29]. This method ensures a volumetric region for safe maneuverability, ideal for robotic applications that need safety when performing movements.

Let *C* be a *n*-dimensional space with all possible robot configurations **q** and let *C*_f_ and *C*_o_ be subsets of *C*, where *C*_f_ is the obstacle-free region and *C*_o_ is the obstacle region in *C* (*C* = *C*_f_ ∪ *C*_o_). A bubble *b* is a volumetric region computed in *C*_f_, which provides safe regions. The region *b* is a *n*−ball and its surface is a (*n* − 1)-sphere in the free configuration space C_f_; it can be defined as

(1) $$b=b\left(q_{c},r\right)=\left\{q:d(q,q_{c})<r\right\},$$

where *r* is the radius of the bubble, *q*_c_ is its the center, and *d*(*q*,*q*_c_) is the metric adopted in the configuration space. In other words, a bubble expands from its center *q*_c_ to the nearest C-obstacle *C_o_* or it can be computed using metrics in the workspace [31].

In this method, the free-space is covered by a set of overlapped bubbles from an initial configuration to a goal configuration in a tree structure called probabilistic foam. The probabilistic foam performs an approximated coverage, similar to some methods based on approximated convex cell decomposition. The foam propagation strategy is analogous to the mechanisms of a breadth-first search as well as wavefront propagation, commonly used in methods based on potential fields.

### 2.1. Foam Propagation

**Figure 2:**
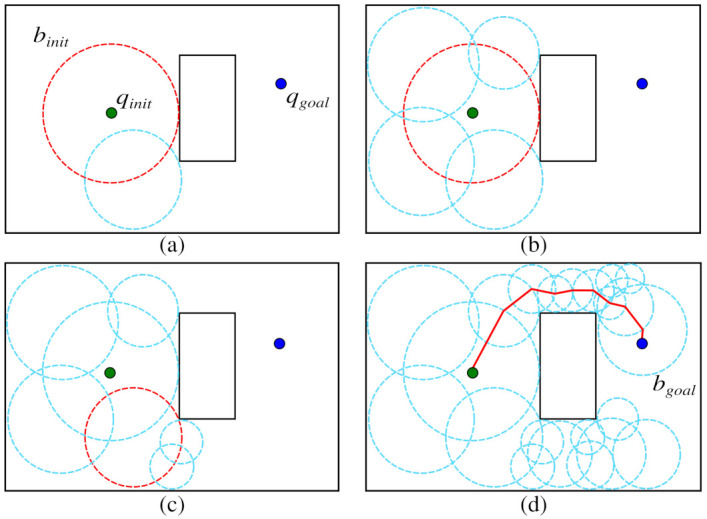
PFM propagation from initial (green dot) to goal configuration (blue dot). (**a**) Initial parent bubble (red circle) and first child bubble. (**b**) Four possible children bubbles. (**c**) New PFM generation with parent bubble selected and children bubbles expanded. (**d**) Probabilistic foam and path found.

Propagation occurs by expanding child bubbles $b_{child}$ on the free surface of parent bubbles $b_{parent}$ (bubbles from the previous generation). The propagation starts from the initial bubble $b_{init}$ (i.e., the bubble centered at the initial configuration). Each parent bubble can expand a maximum of *N* child bubbles, as shown in Equation (2):

(2) $$N=K{\left(\left\lfloor \frac{r}{r_{\min}}\right\rfloor \right)}^{n-1},$$

where $r_{\min}$ is the radius of the smallest allowed bubble and can be estimated by analyzing the width of the passages on the map. The parameter *n* is the dimension of the configuration space and *r* is the radius of the parent bubble. Finally, *K* is a constant that indicates the maximum number of child bubbles allowed for the bubble with radius $r_{\min}$. The process of foam propagation is illustrated in Figure 2.

Child bubbles are expanded over the parent bubble surface during the foam propagation process. However, this expansion only occurs in regions that were not already covered by the foam (see Figure 2a,b). For each generation, all previous child bubbles are selected as parent bubbles; then, new bubbles are expanded (Figure 2c). When some bubble encircles the goal configuration, $b_{goal}$, the propagation finishes (Figure 2d). Finally, the rosary can be found by following the parental relation between the bubbles from $b_{goal}$ to $b_{init}$, and a collision-free path can be extracted by connecting the center of the bubbles, as shown in Figure 1, solving the path planning problem.

In previous studies [29,31,34], the constant *K* was determined by empirical analysis. In this paper, we propose a new approach to determine the value of *K* according to the dimension of the configuration space.

#### Setting the Constant K

The constant *K* is an important parameter of Equation (2), which indicates the maximum number of child bubbles necessary to cover the entire surface area of bubble with radius $r_{\min}$.

**Figure 3:**
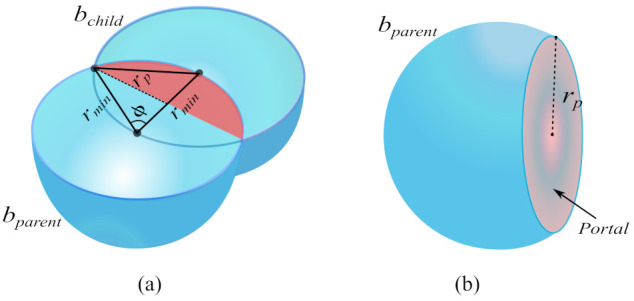
(**a**) Triangle formed between two overlapped bubbles *b_parent_* and *b_child_* with radius *r*_min_. The red region represents the hyperspherical cap. (**b**) Parent bubble and portal region.

Considering the best-case scenario, where the bubbles $b_{parent}$ and $b_{child}$ have an equal radius ($r_{\min}$), the triangle formed between these bubbles (as shown in Figure 3) is equilateral, with all three sides equal to $r_{\min}$. Thus, the colatitude angle of the hyperspherical cap is $\phi =60^{\circ }$ and the radius of the portal region is $r_{p}=\frac{\sqrt{3}}{2}r_{\min}$.

It is possible to obtain *K* as a function of the dimension of the configuration space *n*. This value can be obtained by dividing the surface area $A_{n}$ of a (n−1)-sphere (hypersphere represented by $b_{parent}$ in Figure 3a) by the volume $V_{n-1}$ of the portal, the region is resulted by removing the hyperspherical cap (red area), as can be seen in Figure 3b.

The well-known surface area $A_{n}$ of a (n−1)-sphere, with radius $r_{\min}$ can be expressed by

(3) $$A_{n}\left(r_{\min}\right)=\frac{2\pi^{\frac{n}{2}}}{\Gamma \left(\frac{n}{2}\right)}{r_{\min}}^{n-1},$$

where Γ denotes the gamma function [35]. Based on [36], the volume $V_{n-1}$ of a (n−1)-ball (portal region), with radius $r_{p}$, is defined as

(4) $$V_{n-1}\left(r_{p}\right)=\frac{\pi^{\frac{n-1}{2}}}{\Gamma \left(\frac{n-1}{2}+1\right)}{r_{p}}^{n-1}.$$

Thus, the value of *K*, given dimension *n* of the C-space, can be defined by

(5) $$K=\frac{A_{n}\left(r_{\min}\right)}{V_{n-1}\left(r_{p}\right)}=\left\lceil \frac{2\sqrt{\pi }}{{\frac{\sqrt{3}}{2}}^{n-1}}\;\cdot \;\frac{\Gamma \left(\frac{n+1}{2}\right)}{\Gamma \left(\frac{n}{2}\right)}\right\rceil .$$

**Figure 4:**
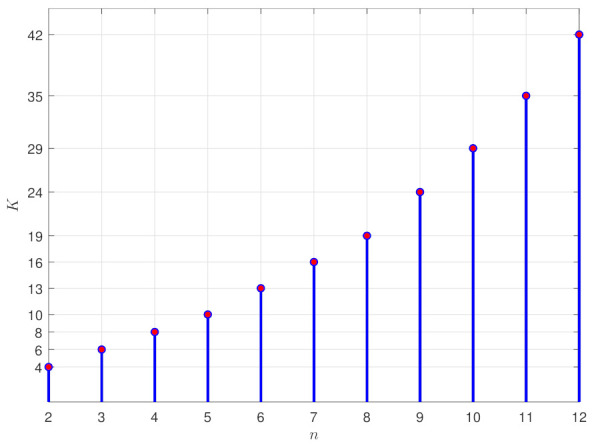
Value of the constant *K* for dimension varying from 2 to 12.

Using Equation (5), it is possible to compute the number of bubbles necessary for a parent bubble with radius $r_{\min}$. Figure 4 shows the value of *K* for the dimension *n* of the C-space varying from 2 to 12.

### 2.2. Pseudocode of PFM

The procedure presented in Algorithm 1 describes the Probabilistic Foam Method. The algorithm receives as input the configurations $q_{init}$ and $q_{goal}$, the minimum radius $r_{\min}$, and the set of obstacles $C_{o}$, and returns the rosary R, from which it is possible to extract the path. The list *F* and the queue *Q* store all bubbles in the foam and the child bubbles for each generation, respectively. The list *F* represents the probabilistic foam itself, and the data structure *Q* has all candidate parent bubbles. When some bubble expands, it is removed from *Q*. The function $\mathrm{expand}\_\mathrm{bubble}(q_{init},C_{o})$ at *line 3* returns the radius of a new bubble. The function $\mathrm{add}(\left\{q_{init},r\right\})$ (*line 4*) stores a bubble (center and radius) in the list *F*.

For each generation, a parent bubble is selected from the queue *Q* (*line 7*); the maximum number of child bubbles *N* is computed (*line 8*); and using the function $\mathrm{surface}\_\mathrm{random}\_\mathrm{config}(q_{p},r_{p})$, a configuration is sampled on the parent bubble surface (*line 10*). If the configuration is not sampled in the interior of another bubble in the foam (verified using the function $\mathrm{int}(q_{i})$ on (*line 11*), a new bubble is expanded (*line 12*). If the radius of the new bubble is greater than or equal to $r_{\min}$, this bubble is stored in lists *F* and *Q*. For each new bubble added to the foam, it is verified if the new bubble encircles the configuration $q_{goal}$ (*line 16*). If it does, the rosary R is extracted from the foam *F* using the function get_rosary() and the algorithm stops, returning success. Otherwise, the current bubble is removed from *Q*, and the next parent bubble will be selected.

The original Probabilistic Foam method described in this section does not present any mechanism that improves the foam propagation strategy to obtain safer and shorter paths or to decrease the processing time of the algorithm. In this way, in the next sections, we present some variants of the original PFM with different propagation approaches.
**Algorithm 1:** Probabilistic Foam Method.
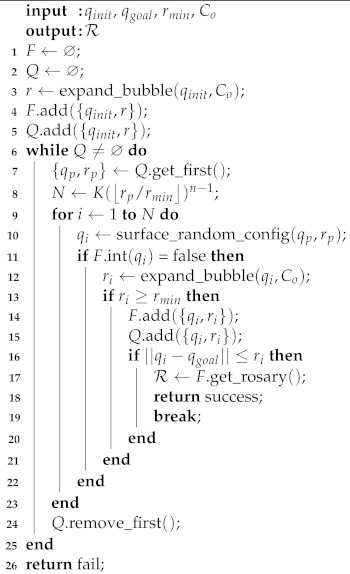


## 3. Variants of Probabilistic Foam

### 3.1. Goal-Biased Probabilistic Foam

**Figure 5:**
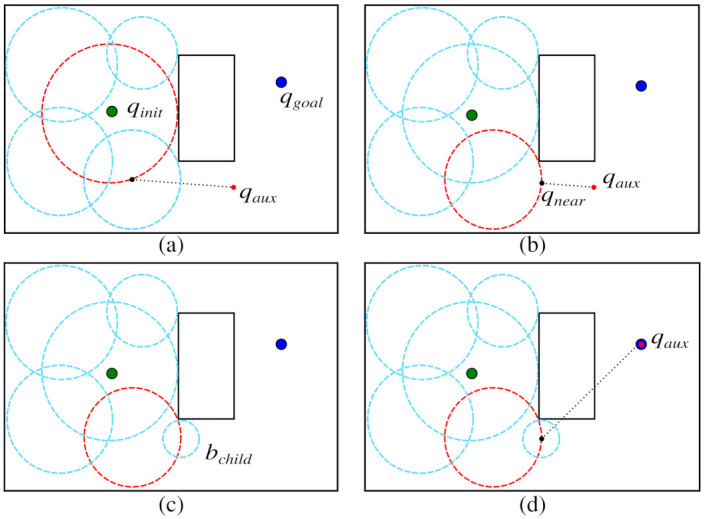
GBPF propagation from initial (green dot) to goal configuration (blue dot). (**a**) *q_aux_* (red dot) is sampled and the nearest parent bubble is selected. (**b**) The configuration *q_near_* is selected. (**c**) A new bubble on *q_near_* is expanded. (**d**) The configuration *q_aux_* is sampled on the *q_goal_*.

Goal-Biased Probabilistic Foam (GBPF) is a variant of the original PFM proposed in [34]. In this algorithm, foam propagation is based on the strategy of expanding the search tree of the RRT-GoalBias algorithm [14], a variant of the classic path planner Rapidly-Exploring Random Tree [13]. The algorithm GBPF usually converges to the goal configuration faster than the original PFM, so the search time is reduced. The main difference between GBPF and the original PFM is that random configurations $q_{aux}$ are sampled in the configuration space, and they guide the propagation of the probabilistic foam. This process is illustrated in Figure 5.

A configuration $q_{aux}$ is sampled in the configuration space (Figure 5a). The parent bubble for this generation will be the one with the center closest to configuration $q_{aux}$. Next, the configuration $q_{near}$ is found on the parent bubble surface (Figure 5b). Then, a new child bubble centered in $q_{near}$ is expanded (Figure 5c). Finally, Figure 5d illustrates how the propagation is biased. There is a small probability (such as 0.05, as suggested by [14]) of sampling the configuration $q_{aux}$ on the $q_{goal}$. In this way, the next parent bubble will be the bubble with the center closest to $q_{goal}$.

The described process is repeated until a child bubble encloses the goal configuration $q_{goal}$, then, the method finds the rosary and the associated path. Algorithm 2 describes the steps of GBPF.
**Algorithm 2:** Goal-biased Probabilistic Foam.
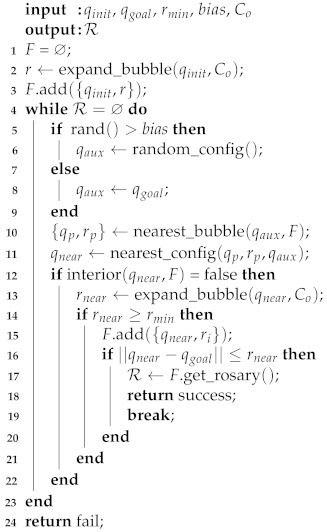


The method described in Algorithm 2 receives as input $q_{init}$, $q_{goal}$, $r_{\min}$, the value of bias, and $C_{o}$, and returns the rosary R. In the same way as described in Algorithm 1, the first bubble, centered in $q_{init}$, is added to the foam *F*. The sampling of $q_{aux}$ occurs on *lines 5–9*. The function rand() returns a uniform random value between [0,1].

The function $\mathrm{nearest}\_\mathrm{bubble}(q_{aux},F)$ returns the bubble (center and radius) to *F* with the nearest center to $q_{aux}$. Additionally, the function $\mathrm{nearest}\_\mathrm{config}(q_{p},r_{p},q_{aux})$ returns the nearest point between the surface of this bubble and the configuration $q_{aux}$ (*lines 10 and 11*). From *line 13* to the end, the method follows the same structure from Algorithm 1. The GBPF method runs until the rosary R is found.

### 3.2. Radius-Biased Probabilistic Foam

Radius-Biased Probabilistic Foam (RBPF) is a new variant of PFM proposed in this work that finds a path considering large passages in the environment, i.e., paths with high clearance from the obstacles. Further, it can be applied to problems where finding a short path is not the main objective. The main difference between RBPF and the original PFM is that the bubbles with a large radius have a higher probability of being selected as parent bubbles. Thus, the probabilistic foam will be biased to propagate faster through the passages with high clearance from obstacles on the map.

The new expanded bubbles are stored in a list Open_List during the RBPF propagation, and for each bubble, a probability $p_{i}$ proportional to its radius is calculated. If $r_{i}$ is the radius of the bubble $b_{i}$ in the foam *F*, its associated probability of being selected is

(6) $$p_{i}=\frac{r_{i}}{\sum_{j=1}^{z}r_{j}},$$

where *z* is the number of bubbles in the list Open_List. This selection strategy is well-known as Roulette Wheel Selection, commonly used in metaheuristic applications, such as the Genetic Algorithm [37,38].

**Figure 6:**
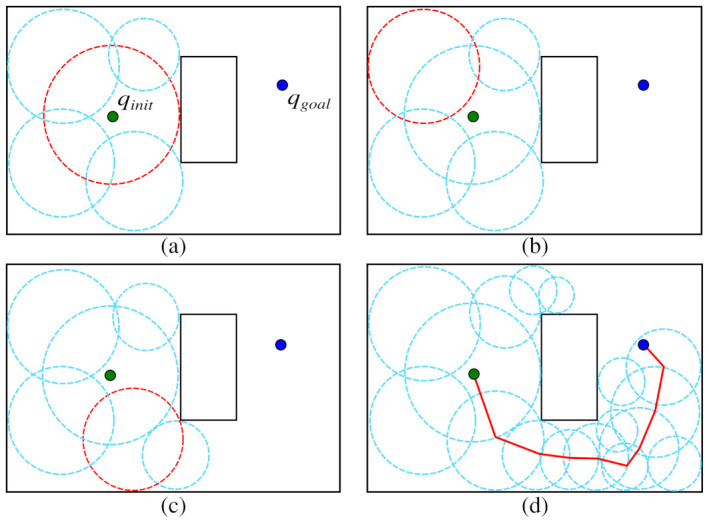
RBPF propagation from *q_init_* (green dot) to *q_goal_* (blue dot). (**a**) Bubbles from the first generation. (**b**) Roulette selects a large parent bubble (red circle) but no child bubble has expanded due to the limited space. (**c**) Roulette selects a parent bubble and a child bubble was expanded. (**d**) The probabilistic foam founds a safe path.

Foam propagation of RBPF is illustrated in Figure 6 for a better understanding of the process. The first generation of the RBPF is very similar to the original PFM (see Figure 2). As shown in Figure 6a, the parent bubble centered in $q_{init}$ (red border) is covered by four child bubbles. These child bubbles are candidates to be parent bubbles for the next generation, i.e., they are stored in the Open_List.

As previously discussed, in RBPF, the parent bubbles with larger radius are selected with high probability using the Roulette algorithm. In Figure 6b, the Roulette algorithm chose the greatest bubble as parent bubble. However, due to the minimum radius rule, no child bubbles were expanded. In the next generation, shown in Figure 6c, another parent bubble was selected, and a new child bubble was expanded. Figure 6d shows that the algorithm found a path with high clearance from the obstacles by propagating the bubbles through the wider passages. The pseudocode of the RBPF method is described in Algorithm 3.
**Algorithm 3:** Radius-Biased Probabilistic Foam.
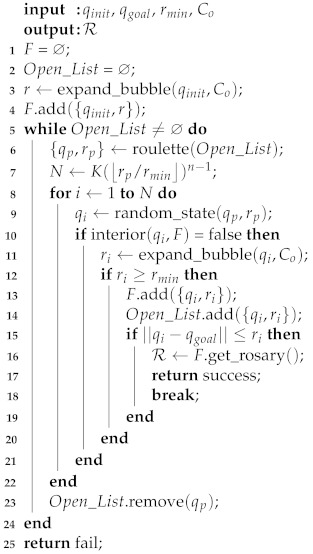


The structure of the Algorithm 3 is very similar to Algorithm 1, which describes the original PFM. The queue *Q* that stores the parent bubbles in Algorithm 1 was replaced to the list Open_List (*line 2*). The main difference between the algorithms is presented on *line 6*, where the function roulette() selects the parent bubbles with probabilities proportional to its radius, as shown in Equation (6). When the child bubbles stop expanding, on *lines 7–22* (procedure explained in Section 2.1 and in most detail in [31]), the parent bubble is removed from Open_List (*line 23*).

### 3.3. Heuristic-Guided Probabilistic Foam

The Heuristic-Guided Probabilistic Foam (HPF) is another variant of the original Probabilistic Foam Method that we propose in this work. This method improves the foam propagation process by adding heuristic information about the goal configuration. This strategy of propagation was inspired by the widely known A* search algorithm [39].

The A* algorithm is an informed heuristic search algorithm that solves search problems in graphs by finding a path with the smallest cost from an initial node to a goal node of a graph [40]. This algorithm combines features from two important algorithms: the Uniform Cost Search and the Greedy Algorithm, ensuring completeness, optimality, and finding solutions in a reasonable amount of time [41].

In the HPF method, the foam propagates through free space by choosing the parent bubble with the smallest cost f(q). The cost of each bubble in the foam is computed by

(7) f(q)=g(q)+h(q).

The function g(q) computes the uniform cost of a bubble in analysis, centered in *q*. The cost *g* is calculated by performing a depth search starting from the bubble under analysis until the initial bubble following the child–parent relationship, and computing the sum of the radius of these bubbles. The function h(q) is a heuristic that estimates the cost between the bubble in analysis and the goal configuration. In this work, this cost is computed using the Euclidean distance. The steps of the variant HPF are described in Algorithm 4.
**Algorithm 4:** Heuristic-Guided Probabilistic Foam.
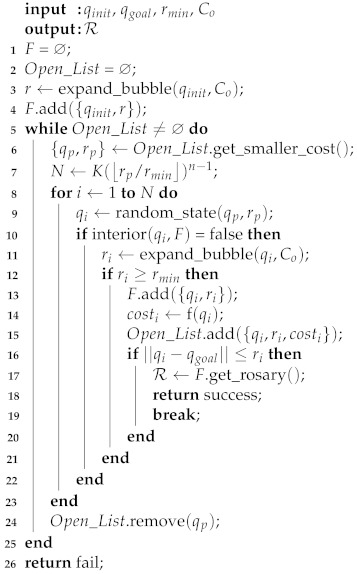


In this new variant, there is a list Open_List to store the parent bubble candidates (center point and radius) and their assigned cost *f*. The method only stops when the list Open_List is empty or when the search finds the rosary R. At each iteration of the algorithm, the bubble with the smallest cost *f* is selected from Open_List by the function get_smaller_cost() (*line 6*). The procedure of expanding child bubbles on the parent bubble surface is similar to the original PFM algorithm. On *line 7*, the maximum number *N* of child bubbles is computed.

The bubbles that met the conditions at *lines 10 and 12* are stored in the foam *F* (*line 13*). Using Equation (7), the costs of the bubbles are computed, and these bubbles and their respective costs are stored in the list Open_List (*lines 14 and 15*). Afterwards, child bubbles are computed and the parent bubble is removed from Open_List (*line 23*).

**Figure 7:**
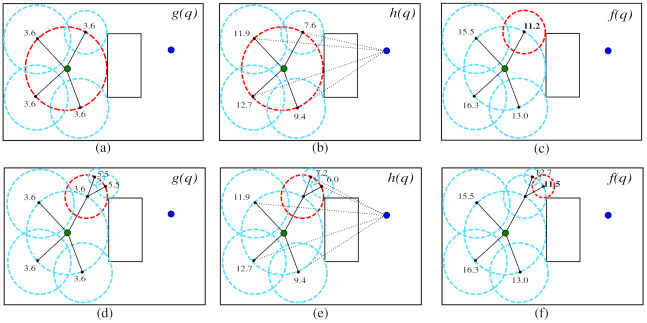
HPF propagation starting from *q_init_* (green dot). (**a**) Computation of *g* cost for all children bubbles on the parent bubble (red circle). (**b**) Computation of *h* cost based on goal configuration (blue dot). (**c**) Computation of *f* cost, and selection of the new parent bubble. (**d**) Cost *g* for the new generation. (**e**) Cost *h* for the new generation. (**f**) Cost *f* for new generation, and selection of the new parent bubble.

An illustration of the propagation process of the HPF is shown in Figure 7.

First, the initial bubble (red bubble) centered in $q_{init}$ (green point) is expanded (Figure 7a). Four child bubbles are expanded on the parent bubble surface and their costs *g* are calculated. In this case, *g* cost is the radius of the parent bubble, g=3.6. In Figure 7b, the heuristic cost *h* is calculated by measuring the Euclidean distance between the bubbles and the $q_{goal}$ configuration (blue point). In Figure 7c, the cost *f* is calculated based on Equation (7). The new parent bubble was chosen by selecting the smallest cost f=11.2; then, a new generation starts.

Two new child bubbles are expanded on the surface of the new parent bubble. The cost *g* of the new bubbles is computed by summing the radius and the *g* cost of the parent bubble, which are equal to 1.9 and 3.6, respectively. Therefore, the value of *g* for the new child bubbles must be equal to 5.5 (1.9+3.6), as shown in Figure 7d. Finally, the new costs *h* and *f* are calculated in Figure 7e,f, respectively, and a new parent bubble is selected.

## 4. Safety Measurement

The studied methods obtain paths with minimum clearance from the obstacles provided by the bubbles. These bubbles are also relevant since they simplify the process of path smoothing, for instance, by imposing safety constraints. However, a rosary usually presents many bubbles with different sizes, which hampers the establishment of a safety measure (i.e., the clearance considering the radius of the bubbles). In this way, we present a metric to measure the path safety based on the Mean Squared Error (MSE) estimator.

Considering that a path is extracted by a sequence of *k* bubbles called rosary and the path safety is measured by considering the radius *r* of these bubbles, the Safety Metric SM for a given path can be calculated by

(8) $$SE=\frac{1}{k}\sum_{i=1}^{k}{\left(r_{i}-r_{\min}\right)}^{2},$$

where $r_{\min}$ is the smallest accepted radius for any bubble in the foam.

The Safety Metric SM estimates how far the radii of bubbles of the rosary are to $r_{\min}$. In this case, the bigger the metric SM, the safer the path.

## 5. Results

This section presents some simulated experiments with the Probabilistic Foam Method and Goal-Biased Probabilistic Foam algorithms, and the two variants of the original PFM proposed in this work, Radius-Biased Probabilistic Foam and Heuristic-Guided Probabilistic Foam. The algorithms were applied to four maps with particular features to analyze the behavior of each method, such as convergence time, path length, number of generated bubbles, and path safety. The developed maps for this work were categorized as narrow, simple, complex, and 3D, as shown in Table 1.

**Figure 8:**
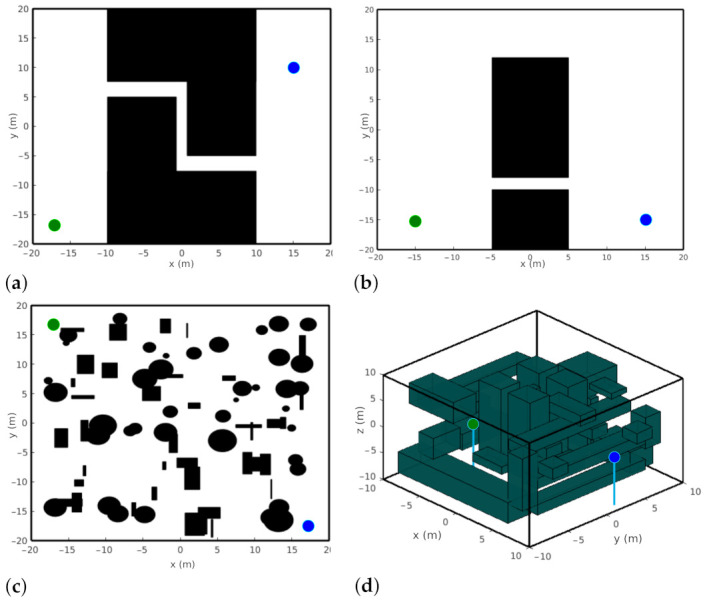
Maps used in simulations, where *q_init_* is the green circle and *q_goal_* is the blue circle. (**a**) Map 1 (Narrow), (**b**) Map 2 (Simple), (**c**) Map 3 (Complex 2D), and (**d**) Map 4 (Complex 3D).

The four maps developed for the simulations are illustrated in Figure 8.

Map 1 (Narrow), shown in Figure 8a, was used to analyze the search time and the number of bubbles for each algorithm to pass through the narrow passage. Map 2 (Simple), shown in Figure 8b, presents two possible routes: the first one is short with a narrow passage, and the second one is long with a wide passage. Map 3 (Complex), shown in Figure 8c, represents a general outdoor environment, which usually has many possible routes for the robot to navigate toward its goal. This map is designed with rectangles and circle-shaped randomly distributed and sized objects. Finally, Map 4 (Complex 3D), shown in Figure 8d, represents a complex outdoor environment with many obstacles and routes in a three-dimensional space. This 3D map was used to analyze the behavior of the methods when the dimension of the configuration space is increased.

These maps present the obstacle regions explicitly represented in C-space. This representation enables the computation of bubbles considering Equation (1). In addition, these regions better illustrate the resulted probabilistic foam, rosaries, and paths. However, for practical experiments, this representation is computationally unfeasible. In this way, new bubbles can be easily computed using information from the workspace without needing the C-obstacle computation, as shown in [30]. Besides, it also allows us to compute bubbles for different robots.

The simulations with the algorithms PFM, GBPF, RBPF, and HPF were performed on a 1.8 GHz Intel Core i7 processor with 8 GB RAM on the Ubuntu 16.04 operating system. The parameters of the algorithms were determined according to the map and were used for all algorithms. The parameter $R_{\min}$ was determined empirically where for Narrow map, $R_{\min}=0.08$; for simple map, $R_{\min}=0.2$; for Complex map, $R_{\min}=0.15$; and for Complex 3D map, $R_{\min}=0.3$. The parameter bias was necessary only for the GBPF algorithm, and was determined as bias=0.05 (as suggested by [14]) for all experiments. Finally, the *K* parameter is determined by Equation (5), according to the dimension of the configuration space. Thus, for the 2D case and 3D case, we use K=4 and K=5, respectively.

**Figure 9:**
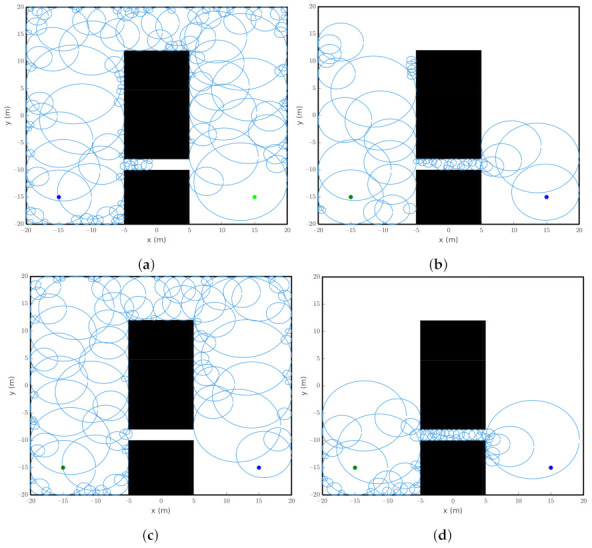
Probabilistic foam generated by the simulations for Map 2 with the algorithms (**a**) PFM, (**b**) GBPF, (**c**) RBPF, and (**d**) HPF.

Simulations were performed to demonstrate the probabilistic foam behavior and some features of the methods. Figure 9 shows the generated probabilistic foam considering the four methods for one simulation using Map 2.

**Figure 10:**
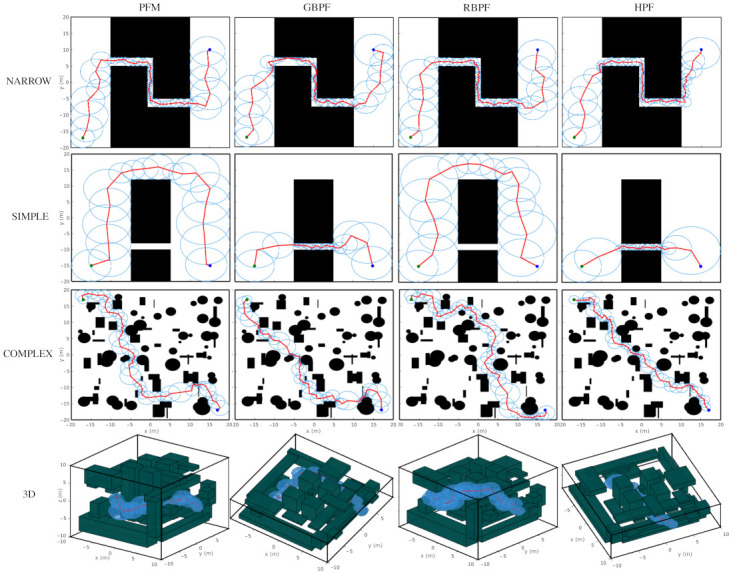
Rosary and paths (from *q_init_*, the green circle; *q_goal_*, the blue circle) found by a simulation with PFM, GBPF, RBPF, and HPF for all maps.

The probabilistic foam generated by PFM (Figure 9a) is dense, i.e., it has a large number of expanded bubbles. Besides, the probabilistic foam generates large bubbles in large passages on the map, resulting in fast propagation in these areas. Thus, the probability of convergence by this route is greater and, consequently, the paths usually have more clearance. On the other hand, this method does not obtain the shortest path. The obtained paths by PFM are shown in Figure 10.

The simulation with GBPF generated a probabilistic foam less dense than PFM, as can be seen in Figure 9b. Our approach inspired in the Goal-biased RRT algorithm enabled the foam of GBPF to propagate fast towards the configuration $q_{goal}$, and generate fewer bubbles. A disadvantage in this randomized approach is that the foam propagates in a disorganized way, generating many irregular bubbles. Additionally, the strategy to choose the parent bubble used in the GBPF makes some small bubbles expand very close to the obstacles. Therefore, some segments of the resulted path can be close to the obstacles even when there is free space available. This configuration can be observed in all paths generated by GBPF, as seen in Figure 10.

Figure 9c shows the probabilistic foam generated by the RBPF algorithm. According to the main feature of this approach, bubbles with a larger radius have a higher probability to be chosen as parent bubbles. This characteristic facilitates fast propagation through passages with high clearance from the obstacles, which generates safer paths, as can be seen in Figure 10. However, this approach must only be applied when the path must pass through the route with higher clearance on the map, since the bubbles will reach narrow areas only when the foam covers all large areas on the map. This implies that the convergence time can be harmed when the configuration $q_{goal}$ is placed on difficult access areas.

Finally, Figure 9d shows the probabilistic foam generated by the HPF algorithm. The strategy of selecting a parent bubble based on costs allows the foam to propagate faster towards the configuration $q_{goal}$, generating few bubbles. An important feature illustrated in Map 2 (Simple) is that the foam propagation was faster through the narrow passages, resulting in a short path. The paths obtained by all methods for all maps are shown in Figure 10.

Considering the stochastic characteristics of the algorithms, some simulations were performed for each algorithm to analyze the processing time (Time), the number of generated bubbles (Bubbles), and the path length (Path). Each algorithm was performed 500 times for 2D maps and 300 times for 3D maps. Table 2 presents the results.

The simulations performed by the Probabilistic Foam method presented the highest number of generated bubbles in all maps. This happened mainly due to the propagation process of PFM, which conducts an approximated coverage of the entire free space. Moreover, PFM does not have any strategies to optimize the search. The results with the GBPF algorithm were the most inconstant. By analyzing the maximum and minimum processing time for the Narrow map, GBPF presented slower and faster simulations compared with the other methods. On average, GBPF presented the shortest processing time for all maps but the Narrow one. For the last three maps, the Radius-biased Probabilistic Foam method presented the longest paths, since its foam propagates first through the wider passages on the map. In the environment with one passage (Narrow map), RBPF was the fastest algorithm on average.

The main feature of HPF is to find paths with the lowest cost, and the results show that HPF found the shortest paths in all maps. For instance, we ran 500 simulations in Map 2 and the resulted path did not pass through the narrow passage in only 18 of them. Besides, GBPF and HPF presented the lowest number of computed bubbles in relation to the other two methods in all maps.

In Map 4, the dimension of the configuration space is 3D and was considered many obstacles in a complex layout. The methods PFM and RBPF presented a very high number of computed bubbles, which results in a high processing time. On the other hand, both GBPF and HPF were not affected by the increase of the configuration space dimension, presenting a high number of expanded bubbles (as well as the Complex map in 2D), but with low processing time.

Notice that the paths are not smooth, which can be a problem when considering a practical application. However, it is possible to apply optimization techniques to smooth the paths obtained from the probabilistic foam methods, as shown in [30], ensuring both safe and smooth paths.

### Measuring the Safety

Considering the same four maps, the values of $r_{\max}$ for maps Narrow, Simple, Complex, and Complex 3D are 4, 5, 4, and 2, respectively. The Safety Metric SM averages for the simulations are presented in Table 3.

The RBPF method presented the best results regarding safety for all maps, as can be seen in Table 3. This result was expected, since this method presents a higher probability to propagate the probabilistic foam through more large passages; obtain rosaries with large bubbles; and consequently, obtain safer paths.

Observing the safety results for the Simple map, there is a clear difference; PFM and RBPF presented high safety values and the methods GBPF and HPF presented the lowest one. The Simple map presents two possible routes, the longer one is safer than the shorter one. Additionally, the rosaries of these methods presented the most regular bubbles, as shown in Figure 10, and due to the low standard deviation and the high max and min values, it is possible to infer that all obtained paths passed through the longer route. On, the other hand, GBPF and HPF presented a high max value and very low min value. Additionally, they have high std values. In this way, it is possible to infer that the obtained paths for these methods passed through both possible routes but, on average, most paths passed through the short passage.

For the Complex map, the GBPF method presented the most unfavorable results. Due to the propagation strategy of GBPF, its rosary usually presents irregular and small bubbles, which means that some segments of the path are close to an obstacle. For both the Narrow and Complex maps, on average, HPF obtained paths safer than PFM and GBPF.

Finally, for the Complex map, the results were similar to the Simple map for the same reason: RBPF and PFM planned paths passed through the larger spaces in the map and GBPF and HPF converged guided by the goal configuration, generating paths that passed through the narrow passages. However, all methods based on probabilistic foam will generate paths with acceptable safety, since they all have rosary with bubbles with at least a minimal acceptable radius. In other words, all methods based on probabilistic foam will generate paths with acceptable safety.

## 6. Conclusions and Future Works

In this paper, we presented some contributions to the robot path planner called the Probabilistic Foam method (PFM). First, an approach to set the value of the constant *K* was formalized, which facilitates understanding of how the method works. Next, we presented three variants of the original PFM: Goal-biased Probabilistic Foam (GBPF), first proposed in [34]; Radius-Biased Probabilistic Foam (RBPF); and Heuristic-Guided Probabilistic Foam (HPF). The last two methods were proposed in this paper. Some simulations were made to analyze the performance of all these methods.

The original PFM and RBPF present similar results, where both find high clearance paths. However, PFM is recommended when free space coverage is necessary, and RBPF can be used when it is most important to find the safest route. The algorithms GBPF and HPF are variants that solve path planning problems by generating a few bubbles and finding paths with low processing times. An advantage of HPF over all variants is the heuristic function that helps it to find shorter paths, maintaining an acceptable safety.

The method RBPF considers only the information of the bubble’s radius as a metric for parent bubble selection. Thus, in future works, we consider investigating metrics that can be used alongside the bubble’s radius to bias the search and find safer paths, decrease the number of computed bubbles, and reduce the searching time.

The new approach to finding the value of *K* is an interesting achievement because the methods PFM, RBPF, and HPF only need to deal with one adjustable parameter, the minimum radius $r_{\min}$ admissible for a given environment.

We noticed an increase in the processing time when the complexity of the environment was increased for some maps. In future works, we intend to investigate some strategies to improve the computational efficiency of the methods, making implementation of the method possible for real-time applications.

## Figures and Tables

**Table 1:** Features of the maps used in the simulations.

Category	Routes	Obstacles	Features
Narrow	1	2	Narrow passage
Simple	2	2	Clearance X Path length
Complex	Many	Many	Outdoor
Complex 3D	Many	Many	3D C-space

**Table 2:** Numerical results of processing time, number of bubbles, and path length for multiple simulations with the algorithms for all maps.

Map	Algorithm	Time (s)				Bubbles				Path (m)			
		Max	Min	Avg	Std	Max	Min	Avg	Std	Max	Min	Avg	Std
Narrow	PFM	2.23	1.02	1.23	0.12	509	386	447.99	21.89	97.14	77.14	85.78	2.80
	GBPF	8.78	0.65	1.85	1.05	454	220	302.34	38.93	109.99	79.37	89.94	5.39
	RBPF	2.68	0.94	1.21	0.25	495	368	428.36	23.72	97.67	78.92	87.09	3.16
	HPF	1.47	1.09	1.21	0.04	458	365	409.49	14.54	89.45	77.67	82.68	2.16
Simple	PFM	0.40	0.16	0.22	0.03	140	88	114.07	9.63	97.58	73.73	83.75	3.92
	GBPF	0.29	0.02	0.06	0.03	110	31	62.88	13.49	111.99	34.69	68.64	26.42
	RBPF	0.30	0.09	0.16	0.03	125	73	100.35	7.06	103.91	78.59	89.10	4.16
	HPF	0.94	0.08	0.15	0.07	136	45	61.04	12.00	87.70	34.49	40.15	8.69
Complex	PFM	13.05	11.29	12.27	0.29	1311	1151	1232.98	27.29	78.93	58.51	66.27	3.88
	GBPF	8.74	0.87	3.19	1.43	944	207	519.66	151.74	89.10	57.47	68.22	5.63
	RBPF	9.92	6.04	8.69	0.63	1221	887	1115.06	59.00	106.79	64.75	79.96	7.19
	HPF	9.38	3.91	6.72	0.84	987	484	751.14	79.97	70.28	57.86	62.40	1.77
3D	PFM	34.32	23.60	29.37	1.71	5638	4195	4979.20	216.85	36.49	19.20	26.86	3.92
	GBPF	10.67	0.18	2.02	1.81	1684	104	582.61	318.40	44.50	18.39	27.69	5.09
	RBPF	43.16	10.91	24.95	4.46	4396	1708	3254.70	510.19	49.98	22.98	36.28	4.39
	HPF	9.17	1.12	3.29	1.21	1395	326	695.91	174.85	24.87	19.74	22.24	0.90

**Table 3:** Average of the safety measurement for the paths obtained by the algorithms PFM, GBPF, RBPF, and HPF.

Map	Algorithm	Safety Metric *SM*			
		Max	Min	Avg	Std
Narrow	PFM	1.7052	0.6646	1.1878	0.2170
	GBPF	2.1576	0.4778	1.2668	0.3237
	RBPF	4.2691	2.6742	3.4347	0.2467
	HPF	3.3945	1.1140	2.3864	0.4088
Simple	PFM	26.6215	16.8881	21.9193	1.9061
	GBPF	25.0479	2.1096	9.1549	5.4033
	RBPF	27.4668	14.5357	22.0905	2.1243
	HPF	26.1911	2.0343	5.8091	4.7272
Complex	PFM	2.2677	1.2036	1.6273	0.1844
	GBPF	1.6130	0.3336	0.7760	0.2239
	RBPF	2.6584	1.4419	1.8995	0.2239
	HPF	1.7052	0.6646	1.1878	0.2170
3D	PFM	2.0272	0.5803	1.2852	0.4990
	GBPF	1.4889	0.0966	0.4066	0.2469
	RBPF	1.9143	1.1021	1.4642	0.2753
	HPF	0.7318	0.1672	0.4121	0.0997

## Data Availability

Data sharing not applicable.

## Abbreviations

The following abbreviations are used in this manuscript:

PFM	Probabilistic Foam Method
GBPF	Goal-Biased Probabilistic Foam
RBPF	Radius-Biased Probabilistic Foam
HPF	Heuristic-Guided Probabilistic Foam
RRT	Rapidly-Exploring Random Tree

## References

[1] Chien R.T., Zhang L., Zhang B. (1984). Planning Collision-Free Paths for Robotic Arm Among Obstacles. IEEE Trans. Pattern Anal. Mach. Intell..

[2] Canny J. (1988). The Complexity of Robot Motion Planning.

[3] Takahashi O., Schilling R.J. (1989). Motion planning in a plane using generalized Voronoi diagrams. IEEE Trans. Robot. Autom..

[4] LaValle S.M. (2006). Planning Algorithms.

[5] Latombe J.C. (1991). Robot Motion Planning.

[6] Latombe J.C. (1991). Approximate Cell Decomposition. Robot Motion Planning.

[7] Khatib O. (1986). Real-Time Obstacle Avoidance for Manipulators and Mobile Robots. Int. J. Robot. Res..

[8] Zhang H., Wang Y., Zheng J., Yu J. (2018). Path planning of industrial robot based on improved RRT algorithm in complex environments. IEEE Access.

[9] Qureshi A.H., Ayaz Y. (2015). Intelligent bidirectional rapidly-exploring random trees for optimal motion planning in complex cluttered environments. Robot. Auton. Syst..

[10] Janson L., Ichter B., Pavone M. (2018). Deterministic sampling-based motion planning: Optimality, complexity, and performance. Int. J. Robot. Res..

[11] Tahir Z., Qureshi A.H., Ayaz Y., Nawaz R. (2018). Potentially guided bidirectionalized RRT* for fast optimal path planning in cluttered environments. Robot. Auton. Syst..

[12] Karaman S., Frazzoli E. (2011). Sampling-Based Algorithms for Optimal Motion Planning. Int. J. Robot. Res..

[13] LaValle S.M. (1998). Rapidly-Exploring Random Trees: A New Tool for Path Planning.

[14] Lavalle S.M., Kuffner J.J. (2001). Rapidly-Exploring Random Trees: Progress and Prospects. Algorithmic and Computational Robotics: New Directions.

[15] Kavraki L., Svestka P., Latombe J.C., Overmars M. (1996). Probabilistic Roadmaps for Path Planning in High-Dimensional Configuration Spaces. IEEE Trans. Robot. Autom..

[16] Volna E., Kotyrba M., Nguyen N.T., Hoang D.H., Hong T.P., Pham H., Trawiński B. (2018). Pathfinding in a Dynamically Changing Environment. Intelligent Information and Database Systems.

[17] Plaku E., Plaku E., Simari P. (2018). Clearance-driven motion planning for mobile robots with differential constraints. Robotica.

[18] Berglund T., Brodnik A., Jonsson H., Staffanson M., Soderkvist I. (2009). Planning smooth and obstacle-avoiding B-spline paths for autonomous mining vehicles. IEEE Trans. Autom. Sci. Eng..

[19] Bhattacharya P., Gavrilova M.L. (2008). Roadmap-Based Path Planning-Using the Voronoi Diagram for a Clearance-Based Shortest Path. IEEE Robot. Autom. Mag..

[20] Xiong C., Chen D., Lu D., Zeng Z., Lian L. (2019). Path planning of multiple autonomous marine vehicles for adaptive sampling using Voronoi-based ant colony optimization. Robot. Auton. Syst..

[21] Geraerts R., Overmars M.H. (2007). Creating high-quality paths for motion planning. Int. J. Robot. Res..

[22] Paliwal S.S., Kala R. (2018). Maximum clearance rapid motion planning algorithm. Robotica.

[23] Zhang H.M., Li M.L., Yang L. (2018). Safe Path Planning of Mobile Robot Based on Improved A* Algorithm in Complex Terrains. Algorithms.

[24] Sent D., Overmars M.H. Motion planning in environments with danger zones. Proceedings of the 2001 ICRA, IEEE International Conference on Robotics and Automation (Cat. No.01CH37164).

[25] Melchior P., Orsoni B., Lavialle O., Poty A., Oustaloup A. (2003). Consideration of obstacle danger level in path planning using A* and Fast-Marching optimisation: Comparative study. Signal Process..

[26] Shahzad K., Iqbal S., Bloodsworth P. (2015). Points-based safe path planning of continuum robots. Int. J. Adv. Robot. Syst..

[27] Lacevic B., Rocco P. (2013). Safety-oriented path planning for articulated robots. Robotica.

[28] Quinlan S., Khatib O. Elastic bands: Connecting path planning and control. Proceedings of the IEEE International Conference on Robotics and Automation.

[29] Silveira Y.S., Alsina P.J. A New Robot Path Planning Method Based on Probabilistic Foam. Proceedings of the 2016 XIII Latin American Robotics Symposium and IV Brazilian Robotics Symposium (LARS/SBR).

[30] Nascimento L.B., Barrios-Aranibar D., Alsina P.J., Santos V.G., Fernandes D.H., Pereira D.S. (2020). A Smooth and Safe Path Planning for an Active Lower Limb Exoskeleton. J. Intell. Robot. Syst..

[31] do Nascimento L.B.P., da Silva Pereira D., Sanca A.S., Eugenio K.J.S., da Silva Fernandes D.H., Alsina P.J., Araujo M.V., Silva M.R. Safe Path Planning Based on Probabilistic Foam for a Lower Limb Active Orthosis to Overcoming an Obstacle. Proceedings of the 2018 Latin American Robotic Symposium, 2018 Brazilian Symposium on Robotics (SBR) and 2018 Workshop on Robotics in Education (WRE).

[32] Santos V.G., Nascimento L.B.P., Fernandes D.H.S., Pereira D.S., Alsina P.J., Araújo M.V. Step modeling and safe path planning for a lower limb exoskeleton. Proceedings of the 2019 19th International Conference on Advanced Robotics (ICAR).

[33] Quinlan S. (1995). Real-Time Modification of Collision-Free Paths. Ph.D. Dissertation.

[34] Nascimento L.B.P., Pereira D.S., Alsina P.J., Silva M.R., Fernandes D.H.S., Roza V.C.C., Sanca A.S. Goal-biased probabilistic foam method for robot path planning. Proceedings of the 2018 IEEE International Conference on Autonomous Robot Systems and Competitions (ICARSC).

[35] Artin E. (1964). The Gamma Function.

[36] Li S. (2011). Concise formulas for the area and volume of a hyperspherical cap. Asian J. Math. Stat..

[37] Qu Y., Zhang Y., Zhang Y. (2017). A Global Path Planning Algorithm for Fixed-wing UAVs. J. Intell. Robot. Syst..

[38] Zeng Z., Sammut K., Lian L., He F., Lammas A., Tang Y. (2016). A comparison of optimization techniques for AUV path planning in environments with ocean currents. Robot. Auton. Syst..

[39] Hart P.E., Nilsson N.J., Raphael B. (1968). A Formal Basis for the Heuristic Determination of Minimum Cost Paths. IEEE Trans. Syst. Sci. Cybern..

[40] Fu B., Chen L., Zhou Y., Zheng D., Wei Z., Dai J., Pan H. (2018). An improved A* algorithm for the industrial robot path planning with high success rate and short length. Robot. Auton. Syst..

[41] Russell S.J., Norvig P. (1995). Artificial Intelligence: A Modern Approach.

